# Changes in the Proteome and Phosphoproteome of *Zea mays* Tissues in Drought Stress Show Plant Tissue Responses from Dehydrins, Carboxylic Acid Metabolism, RNA Splicing and Transcription Factors

**DOI:** 10.3390/proteomes14020025

**Published:** 2026-05-09

**Authors:** Georgina H. Charlton, Cleidiane Zampronio, Andrew R. Bottrill, John Sinclair, Peter M. Kilby, Alexandra M. E. Jones

**Affiliations:** 1 Department of Chemistry, University of Warwick, Gibbet Hill Road, Coventry CV4 7AL, UK; 2 The Molecular Analytical Science Centre for Doctoral Training (MAS CDT), Senate House, University of Warwick, Coventry CV4 7AL, UK; 3 School of Life Sciences, University of Warwick, Gibbet Hill Road, Coventry CV4 7AL, UK; 4 Syngenta Jealott’s Hill International Research Centre, Bracknell, Berkshire RG42 6EY, UK

**Keywords:** maize, corn, phosphorylation, drought, silks, leaves

## Abstract

Background: Maize is a vital crop, supporting 19.5% of global calorie intake. However, maize is vulnerable to even brief periods of drought which substantially reduces seed set and therefore yield. Methods: To identify proteins involved in responses of maize to drought, soluble proteins were extracted from leaf and silk tissues of *Zea mays* and protein abundance and phosphorylation status were quantified relative to well-watered controls. Label-free quantification and phosphopeptide enrichment were applied to the same biological samples and over 300 proteins were identified with significantly different changes. Results: Proteins known to be involved in drought responses were identified, such as the abscisic acid pathway and transcription factors. Of particular interest is a group of dehydrins quantified at both total protein and phosphopeptide levels, permitting insight into stoichiometry. The biological function of dehydrins in the model plant *Arabidopsis thaliana* is known to be regulated by phosphorylation. Conclusions: Translation of protein function from model plant to crops remains highly challenging because genome duplication has created complex sets of orthologous and homologous proteins. By focusing on proteomic changes during crop stress responses, this work enables the identification of known and novel proteins, substantially aiding the transfer of knowledge from model plants to crops.

## 1. Introduction

Drought stress poses a significant and escalating threat to global agriculture, particularly impacting staple crops like *Zea mays* (maize). Maize is a widely grown cereal with an annual production exceeding 1 billion metric tons [1], supporting 19.5% of global calorie intake [2]. Understanding the intricate mechanisms underlying drought tolerance in maize is paramount for developing resilient varieties. This challenge is underscored by the inherent biological differences within plant tissues, particularly between maize leaves and silks. Leaves, as the primary photosynthetic organs, are critical for carbon assimilation, driving growth and yield. In contrast, maize silks exhibit a transient yet functionally vital role in reproduction (and hence yield), primarily characterised by rapid and extensive cell elongation necessary for pollen capture. Abscisic acid (ABA), a key plant hormone, plays a central role in orchestrating adaptive responses to drought, mediating processes from stomatal closure to gene expression changes. Underlying these adaptive responses, protein phosphorylation stands out as a rapid and reversible post-translational modification, acting as a crucial molecular switch that integrates stress signals and fine-tunes the activity of numerous proteins involved in drought perception, signal transduction, and metabolic adjustment.

While *Arabidopsis thaliana* has served as a valuable model for fundamental plant molecular biology, its utility for directly extrapolating drought responses to major crops like maize is limited because *Arabidopsis* is a dicotyledonous C3 plant, whereas maize is a monocotyledonous C4 plant. C4 carbon fixation, with its inherent CO_2_-concentrating mechanism, theoretically confers superior water use efficiency and thus enhanced drought tolerance compared to C3 photosynthesis. Despite this physiological advantage, maize was among the crops most affected by climate change-induced drought in 2023 [3]. This disparity underscores the necessity of targeted research in crops like maize, moving beyond model systems to address the specific challenges faced by vital global food sources.

There are genetic complexities in extrapolating findings from a model plant to a major crop: Maize has a significantly more complex and repetitive genome than *A. thaliana* and possesses numerous orthologs and paralogs for many *Arabidopsis* genes. Orthologs are genes in different species that evolved from a common ancestral gene, retaining similar functions. Paralogs are genes within the same species that arose from gene duplication, often leading to diversified or specialised functions. This extensive gene duplication in maize, a paleopolyploid, means that a single *Arabidopsis* gene involved in a specific drought response might correspond to several maize paralogs. Identifying precisely which of these maize orthologs or paralogs are functionally active and critical under specific drought stress conditions, or in particular tissues, is far from simple. Redundancy, sub-functionalisation, and neo-functionalisation among paralogs add layers of complexity, making direct inference challenging. Furthermore, commercial maize varieties that feed a substantial portion of the global population are predominantly genetically hybrid. These hybrids are selected for traits such as yield stability and stress resilience under local conditions, drawing upon diverse parental lines. However, a significant hurdle for public research is that full genome sequences for most commercial maize varieties are typically not available.

In this study *Zea mays* inbred line B73 was used, which is a parental line for numerous elite commercial hybrids and has contributed to increased corn yields from the 1970s [4]. B73 is the main reference genome sequence for maize, published in 2009 [5]. Drought-stressed leaves and silks were used to determine the relative total protein abundance and the phosphorylation status of soluble proteins. This approach offered a direct window into the functional state and regulatory networks active during drought stress in maize. Focusing on the dynamic proteome can help overcome the challenges posed by the extensive gene duplication in maize and could simplify the identification of functionally relevant isoforms. This protein-centric perspective seeks to bridge the gap between fundamental research in model systems and the practical necessity of developing drought-resilient hybrid maize varieties.

## 2. Materials and Methods

### 2.1. Maize Growth, Droughting and Sampling

Maize, *Zea mays*, B73 plants were grown in the Phytobiology facility at Warwick, using individual 5 L pots per plant; temperature was maintained at 28 °C during the day and 25 °C during the night, with a 16 h day 8 h night cycle. Three replicates of three plants were used for each treatment with separate plants serving as well-watered controls compared to the drought-stressed plants.

Drought stress was imposed by withholding water when plants began to produce pollen, at approximately 65 days after emergence. Drought stress was maintained for 1 week before normal watering was resumed. Control plants were well-watered throughout. The second leaf from the bottom of each plant was harvested and the silks were removed from the cobs at the end of the drought period, flash-frozen in liquid nitrogen and stored at −80 °C. Three drought and three control leaves and silks were harvested from separate plants at the end of the drought stress before re-watering commenced to determine seed set.

### 2.2. Protein Extraction

Maize leaves and maize silks were ground in liquid nitrogen to a fine powder. Buffer (PBS, 500 mM sucrose, 10% glycerol, 20 mM ethylenediaminetetraacetic acid (EDTA), 20 mM ethylene glycol-bis(β-aminoethyl ether)-N,N,N′,N′-tetraacetic acid (EGTA), 50 mM sodium fluoride (NaF), 5 mM β-glycerophosphate, 0.6% polyvinylpyrrolidone (PVP), 10 mM ascorbic acid, 1 mM phenylmethylsulfonyl fluoride (PMSF), 1 mM dithiothreitol (DTT), 1× protease inhibitor cocktail (PIC) (AMSBIO, Cambridge, UK)) was added and samples were vortexed and filtered through Miracloth (Merck, Darmstadt, Germany). Samples were then centrifuged at 20,000× *g* for 30 min at 4 °C. Protein concentration was established using a Bradford assay and protein quality was checked by running 20 µg of protein on a 12% SDS-PAGE gel.

### 2.3. Filter-Aided Sample Preparation Digest

Protein samples at 1 mg/mL in PBS pH 7.4 were transferred into 0.5 mL 10 kDa cut off filter columns (Millipore, Glasgow, UK) and spun at 8000× *g* for 20 min. Samples were washed 3 times with 6 M urea (Merck), centrifuging at 8000× *g* for 20 min each time. Samples were washed 3 times with 50 mM ABC. Samples were incubated with 10 mM Tris(2-carboxyethyl)phosphine (TCEP) (Sigma Aldrich, St. Louis, MO, USA) and 40 mM Chloroacetamide (CAA) (Sigma Aldrich) to reduce disulphide bonds and alkylate cysteines, for 30 min at room temperature. TCEP- and CAA-treated samples were spun through the filter at 8000× *g* for 20 min. The filter was washed 3 times with 50 mM HEPES. A total of 1 µg of trypsin (Promega, Southampton, UK) per 100 µg of protein was added, and samples were left digesting overnight at 37 °C. The peptides were then centrifuged through the filter column for 20 min at 8000× *g*. The filter was washed with water to collect any residual peptides left on the filter. Peptides were desalted using C18 reverse phase tips [6] before removal of acetonitrile using a Concentrator Plus Speed Vac (Eppendorf, Leipzig, Germany).

### 2.4. Phosphopeptide Enrichment

After filter-aided sample preparation (FASP) digestion phosphopeptides were enriched using Titanospere titanium dioxide (TiO_2_) beads (GL Sciences Inc. Tokyo Japan). Peptides in 50 mM HEPES were diluted to 5 mM HEPES with 80% acetonitrile (AcN) and 5% TFA. TiO_2_ beads were diluted to 1 mg in 10 µL of 2,5-dihydroxybenzoic acid (DHB) (Merk) solution at 20 mg/mL and mixed for 10 min at 650 rpm. In total, 2 mg of beads were added for every 1 mg of peptides and were mixed gently with end-over-end mixing for 1 h. This was repeated once. Beads were centrifuged at 2000× *g* for 2 min and the supernatant was removed. A C8 membrane was packed into a 200 µL pipette tip to make a column to support the beads. The beads were added on top of the C8 membrane and washed with 10% AcN; this was centrifuged through at 2000× *g* for 10 min. Beads were then washed with 40% AcN and spun at 2000× *g* for 6 min. Beads were finally washed with 60% AcN and spun at 2000× *g* for 4 min. Peptides were eluted from the beads using 5% ammonium hydroxide (Merk) followed by 15% ammonium hydroxide, 25% AcN and centrifuged at 2000× *g* for 2 min. The ammonium hydroxide and AcN were removed by speed vac for 20 min. Samples were resuspended in 2% AcN 0.1% TFA and sonicated for 15 min before the clean up via C18 stage tip.

### 2.5. For All Proteomic Analysis

Peptide mixtures were resuspended in 2% AcN, 0.1% TFA and were analysed by nano-LC-MS/MS using an Ultimate3000 high-performance liquid chromatography system coupled online to an Orbitrap Fusion Tribrid mass spectrometer (Thermo Fisher Scientific, Loughborough UK). Buffer A consisted of water acidified with 0.1% TFA, while buffer B consisted of acetonitrile with 0.1% TFA and samples were run on a 45 min LC gradient. The peptides were first trapped for 5 min at 10 μL/min with 96% buffer A on a trap (Acclaim PepMap µ-precolumn cartridge 300 µm i.d. × 5 mm, 5 μm 100 Å; (Thermo Scientific, Loughborough UK); after trapping, the peptides were separated by a 50 cm analytical column (Acclaim PepMap RSLC 75 µm i.d. × 50 cm, 2 µm, 100 Å; Thermo Fisher Scientific). To avoid cross-contamination between samples, a wash of 30 min was run between biological replicates (one technical replicate was performed for each sample).

Peptides were ionised using a spray voltage of 2.1 kV and a capillary heated at 275 °C. The mass spectrometer was set to acquire full-scan MS spectra (375 to 1575 m/z) for a maximum injection time set to 150 ms at a mass resolution of 120,000 and an automated gain control (AGC) target value set to 500,000. Accurate mass measurements were made using data-dependent acquisition (DDA). The most intense precursors with charge states between +2 and +6 were selected for fragmentation. HCD fragmentation was performed in the HCD cell, with the readout in the Ion Trap mass analyser (isolation window of 1.2 m/z) and an AGC target value of 5000 with a maximum injection time set to 200 ms and a normalised collision energy of 33%.

### 2.6. Maize Leaf Global Proteomic Analysis

The gradient was 8% to 35% B in 24 min at 250 nL/min. Buffer B was then increased to 80% for the cleaning step. Dynamic exclusion was used with a 20 s window to prevent repeated selection of peptides.

### 2.7. Maize Silk Global Proteomic Analysis

The gradient was 8% to 25% B in 82 min at 250 nL/min. Buffer B was then raised to 35% in 9 min and increased to 90% for the cleaning step. Dynamic exclusion was used with a 45 s window to prevent repeated selection of peptides.

### 2.8. Phosphopeptide Detection

The gradient was 6% to 25% B in 31 min at 250 nL/min. Buffer B was then raised to 37% in 10 min and increased to 80% for the cleaning step. Dynamic exclusion was used with a 25 s window to prevent repeated selection of peptides.

### 2.9. Peptide Assignment and Label-Free Quantification Using Max Quant

Xcalibur RAW files were analysed using Max Quant (MQ) version 2.6.6.0 [7]. Samples were searched using the Uniprot *Zea mays* UNIPROT database consisting of 148,866 protein sequences downloaded on 20 December 2024 (https://www.uniprot.org/uniprotkb?query=maize+B73). Trypsin was set as the digestion enzyme, with up to 2 missed cleavages, and a parent ion mass tolerance of 4.5 ppm. Oxidation of Met was set as a variable modification and Carbamidomethyl of Cys as a fixed modification for all searches. For phosphopeptide enrichments, phosphorylation on S, T, and Y was set as a variable modification. Label-free quantification (LFQ) was used for quantification and the match between runs was used.

The protein groups text file from the MQ search was loaded into Perseus [8], and LFQ intensities for each sample were loaded into the main column. Potential contaminant hits, only identified by site, reverse database and single peptide hits, were filtered out of the data. The data was transformed log_2_X and samples were grouped into treated and control. Proteins with missing values in at least 1 out of 3 reps in one group were removed from the dataset and missing values were replaced from the normal distribution. PCA plots and volcano plots could then be generated.

The phospho (STY) sites text file from the MQ search was loaded into Perseus, and intensity 1, 2 and 3 for each sample was loaded into the main column. Potential contaminant hits and reverse database hits were filtered out of the data. The modification table was expanded, samples were grouped into treated and control, and the data was transformed log_2_X. Peptides with missing values of more than 1 out of 3 reps in one group were removed from the dataset and other missing values were replaced from the normal distribution. PCA plots and volcano plots could then be generated.

## 3. Results and Discussion

### 3.1. Drought Reduces Maize Yield

**Figure 1:**
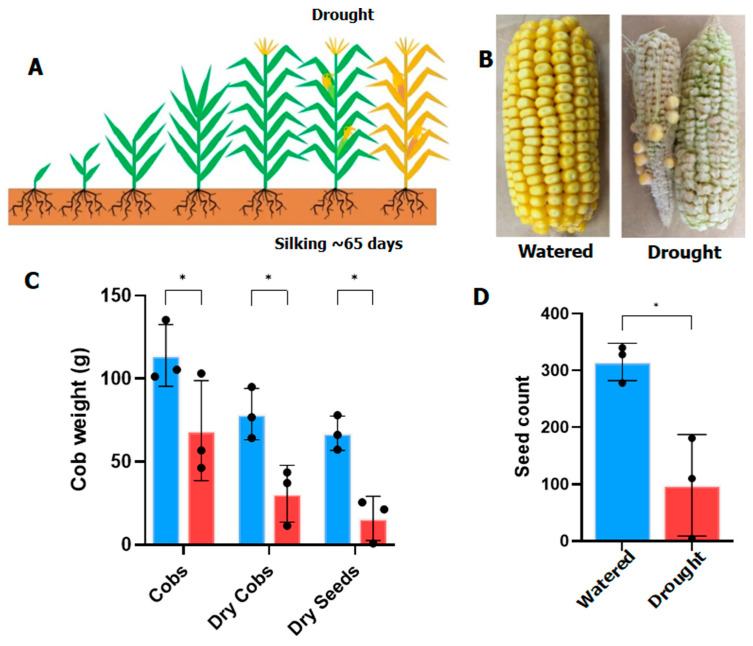
The impact of drought on maize yield for the samples analysed. (**A**). Schematic diagram showing maize growth and drought treatment. (**B**) Typical cobs from well-watered and drought-treated plants. (**C**) Changes to cob weight and (**D**) seed weight and numbers. Well-watered control in blue and droughted samples in red, N = 3. * represents *p* value of <0.05 in the Student’s *t*-test.

Maize is highly vulnerable to drought stress during its flowering cycle. As a monoecious plant, maize has separate pistillate and staminate flowers known as tassels and silks respectively. In this study changes to the proteomes of leaves and silks were compared to gain an understanding of the overall stress on the plants and the direct impact on seed set through drought-damaged silks. Silking maize B73 plants were drought stressed under greenhouse conditions for seven days, during which time the percentage moisture in the soil dropped by at least 30%. Leaves and silks were harvested at the end of the drought period, after which water was reapplied to all plants until cobs were fully matured. The drought-treated cobs showed a significant reduction in dry weight and seed production. Cobs from well-watered plants appeared healthy and full, whereas cobs from droughted plants were much smaller, with numerous kernels missing. (Figure 1).

### 3.2. Leaf and Silk Proteomes Alter Significantly After Drought Stress

**Figure 2:**
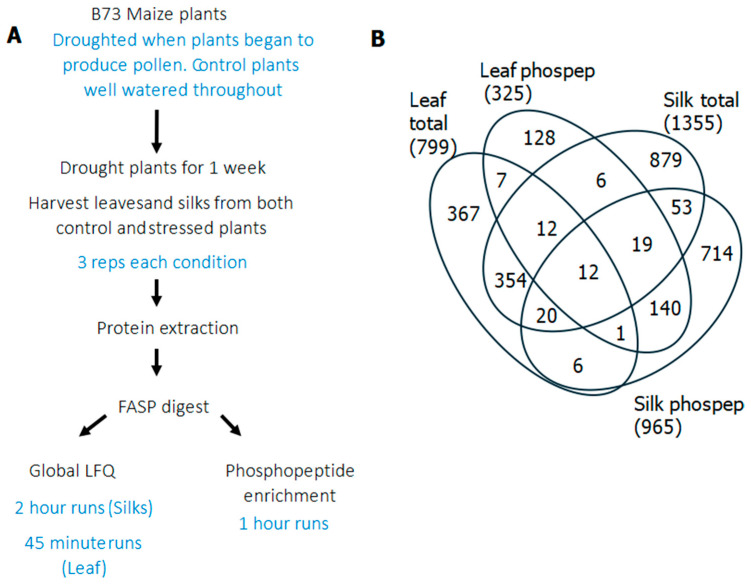
Schematic of workflow (**A**) and summary of proteins and phosphopeptides identified. (**B**) Venn diagram showing commonality between total proteins and phosphopeptides for both leaf and silk samples.

To identify proteins that changed in abundance in maize leaves and silks after drought stress, bottom-up proteomics was used, using LC-MS/MS (Orbitrap Fusion Thermo Scientific). Proteins and phosphopeptides were quantified using MaxQuant and Perseus. Overall, in the four samples (leaf and silk total proteins and phosphopeptides) 2718 proteins were identified and quantified using label-free precursor peak area measurements. The workflow, total numbers of proteins and commonalities between samples are shown in Figure 2 (and Supplementary Figure S2A). The most proteins were identified from the silk samples, likely due to the lack of Rubisco dominating the soluble proteome and the difference in analysis time. The overlap between leaf total protein and phosphopeptides was low at 32 proteins, while the silk total protein and phosphopeptides had an overlap of 104 proteins. In total, 398 proteins were identified in both leaf and silk total protein and 172 proteins were shared between leaf and silk phosphopeptide analyses. As expected from their biology, the proteins identified in these datasets are quite distinct.

**Figure 3:**
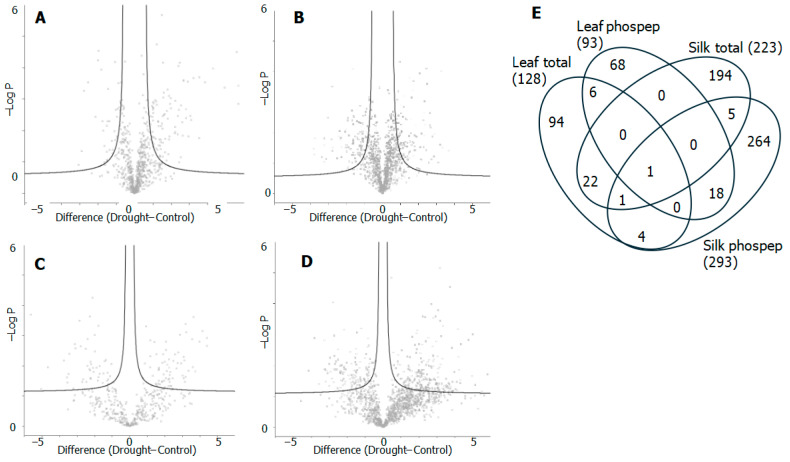
Summary of significantly changing proteins and phosphopeptides. (**A**–**D**) Volcano plots showing the proteins and phosphopeptides which are significantly different between the drought and the control samples; log_2_ fold change is plotted against −logP value. Significance thresholds for phosphopeptides were FDR 0.1 with S0 0.1 and the total protein was FDR 0.05 and S0 0.5. (**A**) Leaf total protein, (**B**) silk total protein, (**C**) leaf phosphopeptides, (**D**) silk phosphopeptides. (**E**) Venn diagram showing commonality between significantly different total proteins and phosphopeptides for both leaf and silk samples. The increased and decreased proteins are not separated in (**E**).

To determine if the proteome and phosphoproteome changed with drought stress, an undirected Principal Component Analysis (PCA) was performed (Supplementary Figure S1). In all cases, the droughted and control samples separate strongly along component one in the PCA accounting for 45% to 69% of the variation between the samples, showing that the drought treatment is changing protein and phosphopeptide abundance. To determine which proteins changed significantly with drought stress, false discovery rate-corrected *t*-tests were applied and are represented as ‘volcano’ plots summarised in Figure 3A–D and Table 1. In total 445 proteins showed an increase in abundance in either total amount or in phosphorylation levels and 273 proteins showed a decrease in total protein abundance or a decrease in the abundance of a phosphopeptide. It should be noted that some proteins showed opposing changes in total protein abundance and phosphorylation state or behaved differently between leaf and silk samples (full details in Supplementary Table S1). The highest number of significantly different proteins was identified in the silk phosphopeptide set (293 proteins), whilst the leaf phosphoproteome had the smallest number of significantly changing proteins (93). There was strikingly little commonality between the four samples in the significantly different proteins and phosphopeptides (Figure 3E and Supplementary Figure S2B).

### 3.3. Dehydrin Proteins Increase in Abundance Under Drought Stress Conditions

Only one protein showed significant changes in all four comparisons (Figure 3, Table 2); dehydrin 3 (C4J477) increased in abundance in the leaf total protein and phosphorylation at T196 while this protein decreased in total abundance in the silks and showed a decreased phosphorylation at the same phosphopeptide. Another dehydrin also showed a significant change (A3KLI1), which increased in leaf total protein and phosphopeptide abundance decreased in silk samples at sites S78 and T69. Dehydrin COR410 (B4G1H1) increased in both leaf phosphorylation and silk phosphopeptides at sites S247 and T194. Dehydrins are also known as late embryogenesis abundant (LEA) proteins and LEA B4F9K0 was identified as significantly increasing in the silk total protein while phosphopeptide abundance of LEA B6SID7 in silks decreased at S3 and Y69 (or S68, as the exact position is uncertain).

**Figure 4:**
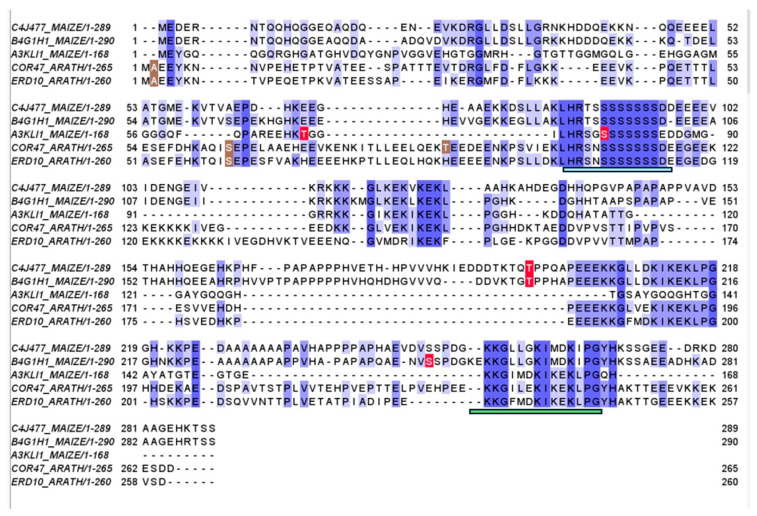
Alignment of three maize dehydrins with two Arabidopsis dehydrins, ERD10 and Cor47. Phosphorylation sites identified in this dataset are marked in red and known PTMs are marked in brown. Shades of blue indicate percent conservation in amino acids. The S-segment is marked with a pale blue underscore. The K-segment with a green underscore.

Late embryogenesis abundant (LEA) proteins, including dehydrins in group II, are highly hydrophilic proteins that accumulate to high levels in plants in response to dehydration stresses such as drought, salinity, and cold [9,10]. They are crucial for survival under stress conditions, acting as molecular chaperones, membrane stabilizers, and osmoprotectants. Fifteen dehydrins are annotated in the *Z. mays* B73 UNIPROT proteome (UP000007305). Dehydrins are characterised by conserved sequence motifs, notably the K-segment (EKKGIMDKIKEKLPG) and the S-segment (a serine tract); the latter is known to be a site of phosphorylation [10]. The sequences of three probable maize dehydrins were aligned with two *Arabidopsis* dehydrins, Cold Regulated 47, COR47, and Early Responsive to dehydration 10, ERD10 (Figure 4). AK3LI1 S78 aligns with the highly conserved S-segment while B4G1H1 T194 aligns with C4J477 T196 in a more variable region that is absent in the other dehydrins.

Dehydrin and LEA gene expression is induced by ABA [9] and they are therefore also known as ABA-responsive proteins. An ABA-responsive protein, K7TFB6, was identified as significantly increasing in the leaf total proteome (but this protein lacks many of the conserved features of the other dehydrins identified in this study).

**Figure 5:**
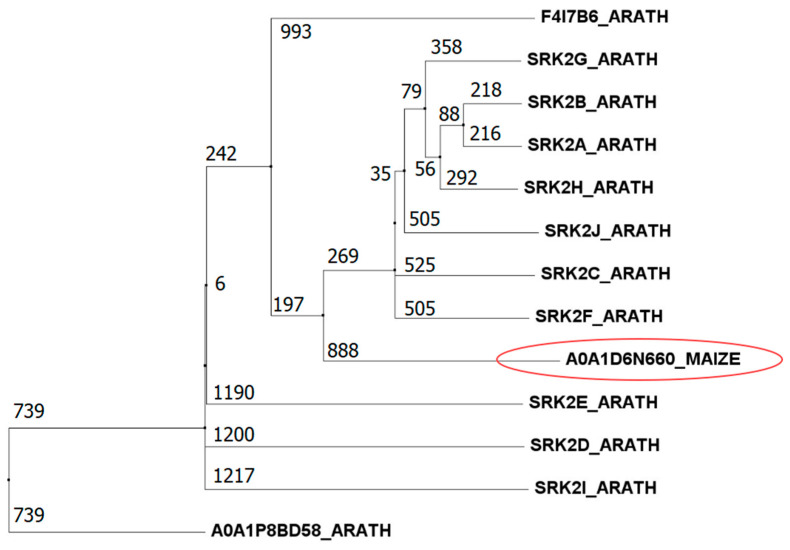
Phylogenetic analysis of the Arabidopsis SnRK2 family with the maize putative SRK2A.

Dehydrins are substrates of both ABA-responsive SnRK2 kinases [10] and of non-ABA-activated SnRK2.10 in *Arabidopsis* [11]. In leaves and silks SRK2A (A0A1D6N660) was observed as significantly increased in phosphopeptide abundance for S158. SRK2A S382 phosphorylation was also observed to increase in silks only while phosphopeptide abundance at S12 (located in the binding site) decreased significantly in silks only. Little is known about SRK2A in maize, but when a Basic Local Alignment Search Tool (BLAST version 2.15.0, NCBI) search was performed, it was found to be 84.1% similar to the ABA response protein SnRK2 in Setaria viridis and the top hit in *Arabidopsis* was to SNF1-related kinase 2.4 (SnRK2.4, also known as OST1-kinase-like 7, 68%). Sucrose non-fermenting 1-related protein kinase 2 (SnRK2) family members are pivotal regulators of plant responses to abiotic stresses, especially drought and salinity [12]. While some SnRK2s (group 3, such as SnRK2.2, SnRK2.3, and SnRK2.6/OST1, in *Arabidopsis*) are strongly activated by ABA and play a core role in ABA signalling. AtSnRK2.4 belongs to group 1, generally considered to be ABA-non-activated kinases, primarily responsive to osmotic stress and other abiotic cues. To determine which group this maize SnRK belongs to, an alignment and then a neighbour-joining phylogenetic tree were made with the 10 *Arabidopsis* SnRK2s (Figure 5). ZmSRK2A clustered more closely to group 1 and group 2 AtSnRK2s and is thus likely to be only weakly or non-responsive to ABA. Despite not being a central player in direct ABA signalling, SnRK2.4 plays a significant and multifaceted role in plant drought responses including regulating root architecture, polyamine biosynthesis, ROS homeostasis, mRNA stability, and developmental senescence [13]. Additionally, group 1 SnRKs are phosphorylated by MAPKs in response to drought stress [14]. A key direct substrate of SnRK2.4 is the transcription factor ABF2 (ABA-response element binding factor 2) which regulates the expression of Arginine Decarboxylase (ADC) in putrescine biosynthesis [15].

### 3.4. Significant Changes in Carboxylic Acid Metabolism in Response to Drought Stress

To determine which processes were significantly altered by drought stress, a GO term enrichment analysis was performed of the proteins identified in this dataset. The category ‘associated with carboxylic acid metabolism’ was the only term substantially enriched in both leaf and silk proteomes for proteins showing significant increase and decrease. The main proteins associated with carboxylic acid metabolism were TCA cycle components and also enzymes associated with hormone metabolism and biosynthesis.

#### 3.4.1. Tricarboxylic Acid (TCA) Cycle

Several proteins and phosphopeptides that are associated with the TCA cycle changed substantially in response to drought stress (Table 3). Phosphoenolpyruvate carboxylase (PEPC) is a crucial enzyme that catalyses the β-carboxylation of phosphoenolpyruvate (PEP) by incorporating bicarbonate to yield oxaloacetate (OAA). Four isoforms of PEPC were identified in the leaf samples and three different PEPC isoforms in silks. In the leaves, the total protein of PEPC (Q43267) decreased significantly in total amount and at three phosphopeptides covering sites (S3, S281, S573). Two other isoforms of PEPC showed an increase in phosphopeptides at a conserved serine residue (A0A1D6I1V6 at S13 and S15 and A0A1D6QPS9 at S7) but a measurement of the protein abundance for those two isoforms in the leaves was not obtained. PEPC (A0A1D6J3B2) decreased significantly in phosphopeptide abundance at S3. In silk total protein, PEPC A0A804P2T6 decreased significantly while A0A804N0Z1 increased in silk phosphopeptides, while PEPC (A0A1D6GB93) increased in phosphopeptide abundance covering sites at T10 and S11. The activity of PEPC is known to be regulated through N-terminal phosphorylation [16]. Other phosphorylation sites were identified on several PEPC isoforms but these did not a show significant change (Supplementary Table S1). Qin et al 2026 [17] and Liu et al 2017 [18] demonstrated that introducing C4-type PEPC into C3 plants (wheat and rice respectively) can significantly enhance their drought tolerance and photosynthetic efficiency. In a previous proteomic analysis of drought-stressed maize, Ref. [19] also identified changes to the phosphorylation status of PEPCs.

In gluconeogenesis, phosphoenolpyruvate carboxykinase (PEPCK) catalyses the reverse reaction to PEPC, using ATP to catalyse the decarboxylation of OAA to PEP. PEPCK is also responsible for decarboxylating C4 acids (such as malate) to release CO_2_ in the bundle sheath cells to enable photosynthesis when stomata are closed. Two isoforms of PEPCK (A0A1D6JWH4, C0P3W9) were identified that showed significant decreases in leaves at several phosphoepetides, but there was no measurement of the total amount of this protein (Supplementary Table S1).

Other TCA-related enzymes that showed significant increases in the leaf proteome include malate synthase (A0A804MED8), citrate synthase (A0A1D6H6F1), and isocitrate dehydrogenase (C0PD27) (Table 3). While other enzymes decreased, malic enzyme (B4F8P6) and 2-oxoglutarate-dependent dioxygenase AOP1 (B4FN73) increased. Sucrose synthase (A0A1D6NT56) showed the largest increase in the leaf total proteome analysis (and another isoform, A0A1D6K2D7, also increased).

#### 3.4.2. Hormones

Several key plant hormones are carboxylic acids or their derivatives: abscisic acid (ABA), indole-3-acetic acid (IAA), jasmonic acid and 1-aminocyclopropane-1-carboxylic acid (ACC) which is the immediate precursor to ethylene. Both ABA- and ACC-related changes were identified in the dataset (Table 4).

1-aminocyclopropane-1-carboxylate oxidase (A0A1D6MIP4) was identified as significantly increased in the leaf proteome and significantly decreased in silk total protein. This enzyme catalyses the final, rate-limiting step in ethylene biosynthesis: the conversion of ACC to ethylene. While ACC synthase (ACS) is often considered the primary regulatory point for ethylene production, accumulating evidence shows that 1-aminocyclopropane-1-carboxylate oxidase (ACO) also plays a critical role in fine-tuning ethylene levels, particularly under stress conditions such as drought [20].

S-Adenosyl-L-Methionine (SAM) is a central metabolite in all living organisms, acting primarily as a universal methyl donor in transmethylation reactions. It is synthesised from ATP and methionine by S-adenosylmethionine synthetase. SAM is the direct precursor for ACC, among many other critical roles. Significant changes to the phosphorylation of a S-adenosyl-L-methionine-dependent methyltransferase (SAM-MTases) A0A1D6NFB3 were identified—a significant increase in phosphopeptide abundance at position S53 in leaves, while S60 increased in silks. Additionally in silks SAM-MTase B4FUW1 decreased in phosphopeptide abundance at site S18. Because SAM has such a central role in methylation it is difficult to attribute a specific function to these observations but these enzymes are not directly in the ACC-to-ethylene conversion pathway.

Three abscisic stress ripening proteins (A0A1D6EB22, A8IK79, B4G0Q9) were identified that changed significantly in leaf and silk total proteomes and showed differential phosphorylation in silks (A0A1D6EB22, S8 decreased and S42 increased, while the total protein increased, A8IK79 decreased in silk total protein, B4G0Q9 increased in silk total protein). These small, plant-specific proteins are hydrophilic and the expression of abscisic stress ripening (ASR) proteins is strongly upregulated by ABA, as well as by various abiotic stresses such as drought, salinity, cold, and even light stress [21]. ASR proteins play a role in stomatal closure [22] but that is unlikely to be relevant in the silk tissues; rather, it could be speculated that as the total protein abundance of dehydrins seems to decrease in silks, perhaps the ASR proteins could fill a similar role as they are also small and hydrophilic.

### 3.5. Notable Differences Between Leaves and Silks

Maize leaves and silks are very different types of tissues, with leaves driving photosynthesis and managing water loss through stomatal closure under drought stress, while silks perform a transient reproductive function and are reliant upon rapid cell growth. Therefore it is not surprising to have observed differences in types of PEPC protein, with the leaves more associated with photosynthesis and different PEPC genes (likely unrelated to photosynthesis) expressed in the silks. The increase in total protein abundance of dehydrins in the leaves might hint at efforts to preserve cells for a longer duration, while the preponderance of transcription factors and splicing factors in the silks suggests that rapid cell growth is being prioritised. Ethylene-related changes (ACC proteins) increased in abundance in the leaf but reduced in the silks, which could be related to stomatal closure and longer-term drought responses in leaf tissues that are not required in the silks.

#### 3.5.1. Splicing Factors

Serine/arginine-rich (SR) splicing factors are a highly conserved family of RNA-binding proteins that play crucial roles in pre-messenger RNA splicing, particularly in the regulation of alternative splicing. Generally SR splicing factors are characterised by one or two N-terminal RNA recognition motifs (RRMs) and a C-terminal arginine/serine-rich (RS) domain [23]. Many highly phosphorylated SR splicing factors were identified predominantly in silks which decreased significantly in phosphopeptide abundance at A0A1D6EWZ5 (S339), A0A1D6HCU4 (S55, S60), A0A1D6N0Q2 (S21, S25) (Table 5). Many other phospho sites on SR proteins were identified but were not significantly altered by drought stress. However in the leaf phosphoproteome A0A1D6H0R9 (T36), B4FEY3 (S239), B6TJ91 (S160) all increased. SR proteins are highly responsive to environmental cues, including drought [24]. Phosphorylation of SR proteins in the RS domain alters their activity, nuclear localisation and modulates splice site recognition and spliceosome assembly [25,26]. SR splicing factors are substrates of the ABA-responsive group 1 SnRK2s [27]. A genome-wide study of alternative splicing in maize drought stress found large induced changes in leaves [28].

#### 3.5.2. Transcription Factors (TF)

Many phosphorylated transcription factors were identified in the silk tissues but the majority did not alter significantly with drought stress (Table 6, Supplementary Table S1). Eight different transcription factors showed significantly different phosphorylation in leaves and silks with no TF in common between these tissues. Two ethylene-responsive transcription factors, AP2-like ethylene-responsive transcription factor AIL1 (A0A1D6HKH6), were identified, which increased in phosphopeptide abundance at S34 in leaves and ethylene-responsive transcription factor ABR1 (B4FQK2), which is also a member of the AP2 family, that increased in phosphopeptide abundance at Y5 in silks. ABR1 is a negative regulator of ABA. Increased phosphopeptide abundance of NF-YB2 was observed in the leaves and this protein enhances drought tolerance by regulating ABA-responsive genes and root architecture [29].

### 3.6. Stoichiometry

One weakness of most phosphorylation enrichment proteomic studies is that it is impossible to determine the stoichiometry of change: Is a phosphorylation site specifically altering or is the total amount of protein changing? Although the overlap between this phospho-dataset and the total proteome is low, some insight can be gained for a few key protein groups. The dehydrins make an interesting case. Dehydrin 3 (C4J477) was observed to increase in both phosphorylation (T196) and total abundance in leaves while in silks both decreased. This implies that the phosphorylation is constitutive and not regulatory. However, in the silks dehydrin COR410 (B4G1H1) was measured in the total protein as not significantly changing but phosphopeptide abundance at site T194 increased significantly. Interesting for dehydrin 1 (A3KLI1) in silks the total amount of this protein was also stable but phosphopeptide abundance decreased at T69 and S78; S78 is in the regulatory S-region. Therefore, these latter phosphorylation sites are more likely to be regulatory.

For other proteins, such as the PEPC and PEPCK, the changes to phosphorylation were consistent with changes to the total amount of protein, where both measurements were obtained. The ABA stress ripening protein (A0A1D6EB22) showed an interesting specificity in silks where the total amount of the protein increased, as did the abundance of phosphoeptides covering site S42 but phosphorylation at S8 decreased. ASR3 is not known to be regulated by phosphorylation but S8 could be a promising candidate site for future research.

Most previous proteomic work on maize during drought has focused on the leaves [19,30,31,32,33,34] or kernels [35,36,37] using other varieties of maize and different treatment times: Ali et al. [30] used *Z. mays* cv. Borderking to identify the impacts of drought by withholding water for 40 days on the leaf proteome. Hu et al. [19] used maize variety ZD958 to identify phosphopeptides that changed in abundance after PEG-stimulated drought stress at the 5 leaf stage of growth. Kim et al. [31] used the inbred maize line KS140 to identify leaf proteins after drought stress at stages V5 to V6. Li et al. [32] used the hybrid Shaanke 9 to examine the impact of mild and severe drought stress at the 6 leaf stage. Zenda et al. [34] used maize lines YE8112 and MO17 to look at drought stress in seedlings.

Despite the differences in varieties, stress and tissues used in these studies and the work presented here, some of the same protein families were identified as increasing in abundance after drought stress. These were: phenylalanine/tyrosine ammonia-lyases and sucrose synthase [30], phosphoenolpyruvate carboxylase, serine-threonine kinases and arginine/serine-rich splicing factors [19], an abscisic stress ripening protein [31]. Studies [32,34] have low overlap with the proteins identified in this study.

Three publications examined the impact of drought on maize kernel proteome using drought resistant compared to drought susceptible varieties, ND476 vs. ZX978 [35,37] and YE8112 vs. MO17 [36]. A slight similarity to the proteins identified in this study was an increase in abundance of some stress-related proteins [35], proteins associated with oxidative phosphorylation pathways [36], and an ABA-responsive RAB17 protein [36] that is probably similar to the dehydrin 1 identified in this work.

Most similar to the work presented here is Vu et al. [33], in their analysis of leaf phosphoproteins and total protein abundance after reduced watering (to 62.5% of well watered controls) for 21 days, but the variety of maize was different (B104). In the Vu study, 234 differentially abundant proteins were identified from leaves (at stage 7); functionally these proteins showed an over representation in carbohydrate metabolism and chromatin remodelling. There are several commonalities with the proteins identified in this study including lipoxygenases, keto reductase family 4 member and fructose-biphosphate aldolase [33].

Regarding the proteomic analysis of maize silks, the study by Agostini et al. [38] is also similar to the work presented here in using maize B73 and in examining the silk total proteome, but the treatment was during resistance to Fusarium infection after priming induced by Trichoderma root colonisation. Their key results focused on cell wall reinforcement and an increase in flavonoid and lignin content in maize silks after activation of induced systemic resistance.

### 3.7. Concluding Consideration of Complexity and Limitations

The dramatic difference in proteins identified in leaf and silk tissues emphasizes the flexibility and dynamic nature of the maize proteome. This study shows the need to isolate and characterise tissues separately to capture and understand the complexity of the proteome. Even in an inbred line such as B73, the genome is still highly complex with substantial duplication and divergence of genes. Accounting for splice variants and post-transcriptional processing of proteins the diversity of proteoforms resulted in a UNIPROTKB protein database that contained nearly 150,000 protein entries using up to three splice variants per gene. As this was a peptide-based study all protein identifications were inferred and this resulted in the majority of proteins and phosphopeptides being assigned to multiple records. To facilitate analysis, the first recorded number assigned by Perseus was used to compare proteins and peptides between samples. In Supplementary Table S1 the splice variant number is shown in the protein name and all potential records are displayed in the ‘FASTA header’ column.

A limitation of peptide-based phosphoproteomics is that many protein assignments rely on a single peptide, thereby substantially raising the false positive discovery rate. This was compensated for by focusing on phosphopeptides that passed scoring thresholds and were observed in all three replicates.

Regarding proteoforms, no post-transcriptional processing of the protein sequence (cleavage of pro-propeptides or localisation motifs) could be revealed using current proteomic technology. A fundamental limitation of peptide-based proteomics is that for proteins identified with several phosphorylation sites, the nature of the data precludes the determination of several co-existing proteins that are mono-phosphorylated at different positions, or any combination of the same protein multiply phosphorylated. For simplicity, data is presented as if only one maximally phosphorylated protein existed.

## Figures and Tables

**Table 1:** Summary of proteins and phosphopeptides that altered significantly in abundance with drought stress.

Sample	Sig. Increase Proteins (Phos-Peptides)	Sig. Decrease Proteins (Phos-Peptides)
Leaf Total	91	37
Leaf Phos	62 (70)	32 (53)
Silk Total	110	113
Silk Phos	202 (244)	101 (123)

**Table 2:** Summary of changes in proteins and phosphorylation of dehydrins and related proteins that altered significantly in abundance with drought stress.

Protein	Name	Phos Site	Significant Fold Changes in Leaves (log2)	Significant Fold Changes in Silks (log2)
A3KLI1	Dehydrin 1	S78 T69 total	5.67	−2.54 −2.50
B4G1H1	Dehydrin COR410	S247	3.74	
		T194		2.41
C4J477	Dehydrin 3	T196 total	2.15 4.29	−0.78 −1.93
B6SID7	Late embryogenesis abundant protein	S3	4	−4.15
		S68		−2.67
		Y69 (or S68)		−3.15
B4F9K0	Late embryogenesis abundant protein	total		1.04
K7TFB6	ABA-responsive protein	total	2.82	
A0A1D6N660	Serine/threonine-protein kinase SRK2A	S12		−1.36
		S158	2.24	4.32
		S382		2.39

**Table 3:** Proteins associated with the TCA cycle that were altered significantly in abundance with drought stress.

Protein	Name	Phos Site	Significant Fold Changes in Leaves (log2)	Significant Fold Changes in Silks (log2)
A0A1D6GB93	Phosphoenolpyruvate carboxylase 3	T10 S11		3.43 2.71
A0A1D6J3B2	Phosphoenolpyruvate carboxylase 3	S3	−3.74	
A0A1D6QPS9	Phosphoenolpyruvate carboxylase	S7	3.55	
A0A804N0Z1	Phosphoenolpyruvate carboxylase	S213		1.84
A0A804P2T6	Phosphoenolpyruvate carboxylase	total		−1.68
Q43267	Phosphoenolpyruvate carboxylase	S3 S281 S573 total	−3.43 −2.47 −1.81 −0.94	
A0A1D6JWH4	Phosphoenolpyruvate carboxykinase	S111 T112 S59 S133 S51 S47 total	−1.21 −2.11 −4.66 −2.22 −2.27 −2.41 −2.55	
A0A1D6PEH7	Phosphoenolpyruvate carboxykinase	T51 S47	−2.27 −2.79	
C0P3W9	Phosphoenolpyruvate carboxykinase	S51 T59 S55	−3.21 −2.27 −2.49	
A0A804MED8	Malate synthase	total	2.60	−3.24
A0A1D6H6F1	Citrate synthase	total	2.63	
C0PD27	Isocitrate dehydrogenase	total	1.66	
B4F8P6	Malic enzyme	S617 total	−1.26 −0.84	
B4FN73	2-oxoglutarate-dependent dioxygenase AOP1	total	−1.47	1.13
A0A1D6NT56	Sucrose synthase	S169 total	1.66 5.68	
A0A1D6K2D7	Sucrose synthase	S11 total	3.22 2.83	
A0A1D6P848	Sucrose synthase	total		0.99

**Table 4:** Proteins associated with hormone biosynthesis and metabolism that were altered significantly in abundance with drought stress.

Protein	Name	Phos Site	Significant Fold Changes in Leaves (log2)	Significant Fold Changes in Silks (log2)
A0A1D6MIP4	1-aminocyclopropane-1-carboxylate oxidase	total	2.48	−1.49
A0A1D6NFB3	S-adenosyl-L-methionine-dependent methyltransferase	S53	4.41	
		S60		1.49
B4FUW1	S-adenosyl-L-methionine-dependent methyltransferase	S18		−1.52
A0A1D6EB22	Abscisic acid stress ripening 3	S8 S42 total	2.21	−1.67 2.20 1.45
A8IK79	Abscisic acid stress ripening 5	total		−2.83
B4G0Q9	Abscisic acid stress ripening 2	total		1.74

**Table 5:** Proteins associated with RNA splicing that were altered significantly in abundance with drought stress.

Protein	Name	Phos Site	Significant Fold Changes in Leaves	Significant Fold Changes in Silks
A0A1D6EWZ5	Serine/arginine-rich splicing factor SR45a	S339		−0.77
A0A1D6HCU4	Serine/arginine-rich splicing factor RSZ22	S55 S60		−1.37 −1.54
A0A1D6N0Q2	Serine/arginine-rich splicing factor SR45	S21 S25		−2.23 −1.84
A0A1D6H0R9	Splicing factor 3B subunit 1	T36	2.44	
B4FEY3	Arginine/serine-rich splicing factor RS2Z35	S239	2.20	
B6TJ91	Splicing factor, arginine/serine-rich 7	S160	2.28	

**Table 6:** Transcription factors that altered significantly in abundance with drought stress.

Protein	Name	Phos Site	Significant Fold Changes in Leaves	Significant Fold Changes in Silks
A0A1D6HKH6	AP2-like ethylene-responsive transcription factor AIL1	S34	0.94	
A0A1D6IHZ6	Putative WRKY transcription factor 20	S16	1.64	
A0A1D6K5M3	Nuclear transcription factor Y subunit B-2	S20	1.18	
A0A1D6M0K2	Transcription factor IIA alpha/beta subunit	T141	−1.42	
B4FQK2	Ethylene-responsive transcription factor ABR1	Y5		3.31
B6SV05	GRAS family transcription factor	S280		−1.54
B6T6H6	Transcription factor bHLH130	S189		0.61
B6UEP1	BZIP transcription factor	S11		2.00

## Data Availability

The mass spectrometry proteomics data have been deposited to the ProteomeXchange Consortium via the PRIDE [39,40] partner repository with the dataset identifier PXD061787.

## Supplementary Materials

The following supporting information can be downloaded at: https://www.mdpi.com/article/10.3390/proteomes14020025/s1, Figure S1: (A–D) PCA plots of the phosphopeptides identified from maize leaf and silk tissues. The PCA plot shows separation between drought (red) and control samples (blue) as a percent of total variation. (A) Leaf total protein (B) Silk total protein (C) Leaf phosphopeptides (D) Silk phosphopeptides; Figure S2: UpSet diagrams showing the numbers of unique and shared proteins in each sample type. (A) All proteins and phosphopeptides identified in this study. (B) Proteins and phosphopeptides that show significant change (regardless of increase or decrease). Table S1: List of all proteins and phosphopeptides identified in all samples with normalised intensity.
